# Surgical outcomes in gastroenterological surgery in Japan: Report of the National Clinical Database 2011–2019

**DOI:** 10.1002/ags3.12462

**Published:** 2021-04-09

**Authors:** Shigeru Marubashi, Arata Takahashi, Yoshihiro Kakeji, Hiroshi Hasegawa, Hideki Ueno, Susumu Eguchi, Itaru Endo, Takanori Goi, Akio Saiura, Akira Sasaki, Shuji Takiguchi, Hiroya Takeuchi, Chie Tanaka, Masaji Hashimoto, Naoki Hiki, Akihiko Horiguchi, Tadahiko Masaki, Kazuhiro Yoshida, Mitsukazu Gotoh, Hiroyuki Konno, Hiroyuki Yamamoto, Hiroaki Miyata, Yasuyuki Seto, Yuko Kitagawa, the National Clinical Database

**Affiliations:** ^1^ The Japanese Society of Gastroenterological Surgery Tokyo Japan; ^2^ Department of Health Policy and Management School of Medicine Keio University Tokyo Japan; ^3^ Department of Healthcare Quality Assessment Graduate School of Medicine The University of Tokyo Tokyo Japan

**Keywords:** gastroenterological surgery, NCD, research report, surgical outcome, treatment outcome

## Abstract

**Background:**

We aimed to present the 2019 annual report of the gastroenterological section of the National Clinical Database (NCD).

**Methods:**

We reviewed 609,589 cases recorded in 2019 and 5,029,764 cases registered from 2011 to 2019 for the 115 selected gastroenterological surgical procedures.

**Results:**

The main features of gastroenterological surgery in Japan were similar to those described in the 2018 annual report, namely, that 1) operative numbers gradually increased in all procedures, except gastrectomy and hepatectomy, which decreased in these years; 2) in all eight major gastroenterological surgeries, the age distribution tended toward older patients; 3) the morbidity of esophagectomy, hepatectomy, and pancreaticoduodenectomy increased, but mortality was minimized in all procedures; 4) all eight major gastroenterological procedures have increasingly been performed under laparoscopy; and 5) board‐certified surgeons were increasingly involved. These trends in recent years were more prominent in 2019.

**Conclusions:**

Thanks to the continuous cooperation and dedication of the surgeons, medical staff, and surgical clinical reviewers who registered the clinical data into the NCD, it is possible to understand the comprehensive landscape of surgery in Japan and to disclose new evidence in this field. The Japanese Society of Gastroenterological Surgery will continue to promote the value of this database and encourage the use of feedback and clinical studies using the NCD, now and in the future. Generating further approaches to surgical quality improvement are important directions for future research.

## Graphical abstract

As the annual report of NCD 2019, data of gastroenterological surgery from 2011 to 2019 in Japan were summarized, and the trends in the 115 gastroenterological procedures and eight major gastroenterological surgeries were reported.

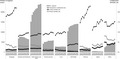

## 1 INTRODUCTION

The National Clinical Database (NCD) has been recognized as the largest and best‐organized nationwide surgical registry in Japan and has become indispensable for surgeons, patients, and the healthcare system of Japan. The NCD was established in 2010 and started its data registration in 2011.^1^, ^2^, ^3^, ^4^ As of January 14, 2021,^5^ 5404 facilities have enrolled in the NCD and approximately 1,500,000 cases have been registered every year, constituting more than 95% of all surgical cases in Japan.^2^

The NCD comprises the members of surgery‐related societies^5^ including the Japan Surgical Society (JSS), the Japanese Society of Gastroenterological Surgery (JSGS), and the Japanese Society of Hepato‐Biliary‐Pancreatic Surgery (JSHBPS), and clinical data are stratified into three levels^3^: 1) basic common variables, 2) subspecialty variables such as those in 115 selected gastroenterological surgeries or eight major gastroenterological surgeries, and 3) more specialized variables such as those in the Hepato‐Biliary‐Pancreatic surgery. Data in the gastroenterological section of the NCD are based on the JSGS’s definition of the variables and include 115 gastroenterological operative procedures considered important for the board certification system. Eight major gastroenterological surgeries were selected among these 115 procedures (esophagectomy [ESO], distal/total gastrectomy [DG/TG], right hemicolectomy [RHC], low anterior resection [LAR], hepatectomy [HEP], pancreaticoduodenectomy [PD], and surgery for acute diffuse peritonitis [ADP]); data on detailed variables including preoperative laboratory findings, comorbidities, and postoperative complications were required.^1^, ^6^, ^7^, ^8^

Gastroenterological surgical procedures are also classified into three groups according to their technical difficulty; low, medium, and high degree of difficulty. Some of the newly approved high‐difficulty procedures, such as laparoscopic major hepatectomy and laparoscopic and robot‐assisted pancreatoduodenectomy, are required to be registered preoperatively in the NCD for health insurance to be authorized by the Ministry of Health, Labour and Welfare in Japan.

Thus, the importance of the NCD has been increasing rapidly as a clinical database and a means for controlling the quality of new surgical procedures. The NCD is also important as it provides medical staff and societies with rigorously collected data for quality improvement of surgery, feedback about surgical outcomes as risk calculators for morbidities^9^, ^10^, ^11^, ^12^, ^13^, ^14^, ^15^, ^16^, ^17^ and mortality,^17^, ^18^, ^19^, ^20^, ^21^, ^22^, ^23^, ^24^, ^25^ data on the comprehensive surgical landscape via the NCD website,^1^, ^5^ and data gathered from various clinical studies.

This report intends to outline the current situation and trends to understand the standpoint of and to elucidate the future directions of gastroenterological surgery in Japan using data from the gastroenterological section of the NCD. Previously, the annual reports of the NCD were published on data from 2011 to 2018, and more than 609,589 cases were newly registered in 2019. We describe the most important findings from the data about gastroenterological surgery in the NCD between 2011 and 2019.

## 2 SUBJECTS AND METHODS

As previously reported,^6^, ^7^, ^8^ the subjects were patients who collectively underwent the 115 surgical procedures stipulated by the “Training Curriculum for Board‐Certified Surgeons in Gastroenterology” and had their surgical data recorded from 2011 to 2019 in the NCD system. Data of basic and perioperative variables were collected for these cases, as described previously.^1^, ^3^, ^8^ Basic common variables were designed for JSS‐level data, comprising age, gender, preoperative and final diagnosis, date of procedure, surgical procedure (NCD code), operator and assistants, emergent or elective surgery, and participation of anesthesiologists. Subspecialty variables, such as the TNM classification for malignant diseases, Clavien–Dindo (C–D) classification^26^, ^27^ of postoperative complications, date of discharge or death, and 30‐day and in‐hospital mortality were collected for the 115 selected surgical procedures. Additionally, detailed laboratory data, preoperative comorbidities and functional status, and types of postoperative morbidities were recorded for the eight major gastroenterological procedures defined above.

Postoperative complications of C–D grade III or greater were defined as severe complications. Anonymous data of the board‐certified gastroenterological surgeons of the JSGS were transferred into the NCD and linked with each procedure to elucidate the participation of board‐certified surgeons.

Data were extracted in a secure system without external connection and basic statistical analysis were carried out by NCD statistic experts, and the number of surgical cases and the mortality rates related to the selected 115 gastroenterological operative procedures were calculated, as well as those for the eight major operative procedures from 2011 to 2019. The incidence of participation of board‐certified surgeons as the primary surgeons or assistants in the eight major gastroenterological surgeries was also calculated.

The NCD system is modified annually to adapt to the change in operative procedures, new surgical techniques, or fit the definition and choices of variables better. In 2019, over 50 modifications were made, including the addition of six new variables and the modification of over 40 parts in the definition and other sections of gastroenterological surgery in NCD.

The following points need to be considered in the interpretation of the data reported here: 1) As a maximum of eight operative procedures can be recorded per case in the NCD, the total number of surgeries in the results describing the 115 gastroenterological surgical procedures for the board certification system does not represent the actual number of surgical cases; 2) Cases with errors in patient age, sex, and postoperative 30‐day status were excluded; 3) Cases in which several operative methods were performed simultaneously were recorded according to all operative methods; 4) Postoperative 30‐day mortality included all cases of mortality within 30 days after surgery, regardless of pre‐ or postdischarge status. The calculation of operative mortality included all patients who died during hospitalization, including hospital stays up to 90 days, and any patient who died after hospital discharge within 30 days of the operative date.

## 3 RESULTS

### 3.1 The 115 selected gastroenterological operative procedures in the “Training Curriculum for Board‐Certified Surgeons in Gastroenterology”

**FIGURE 1:**
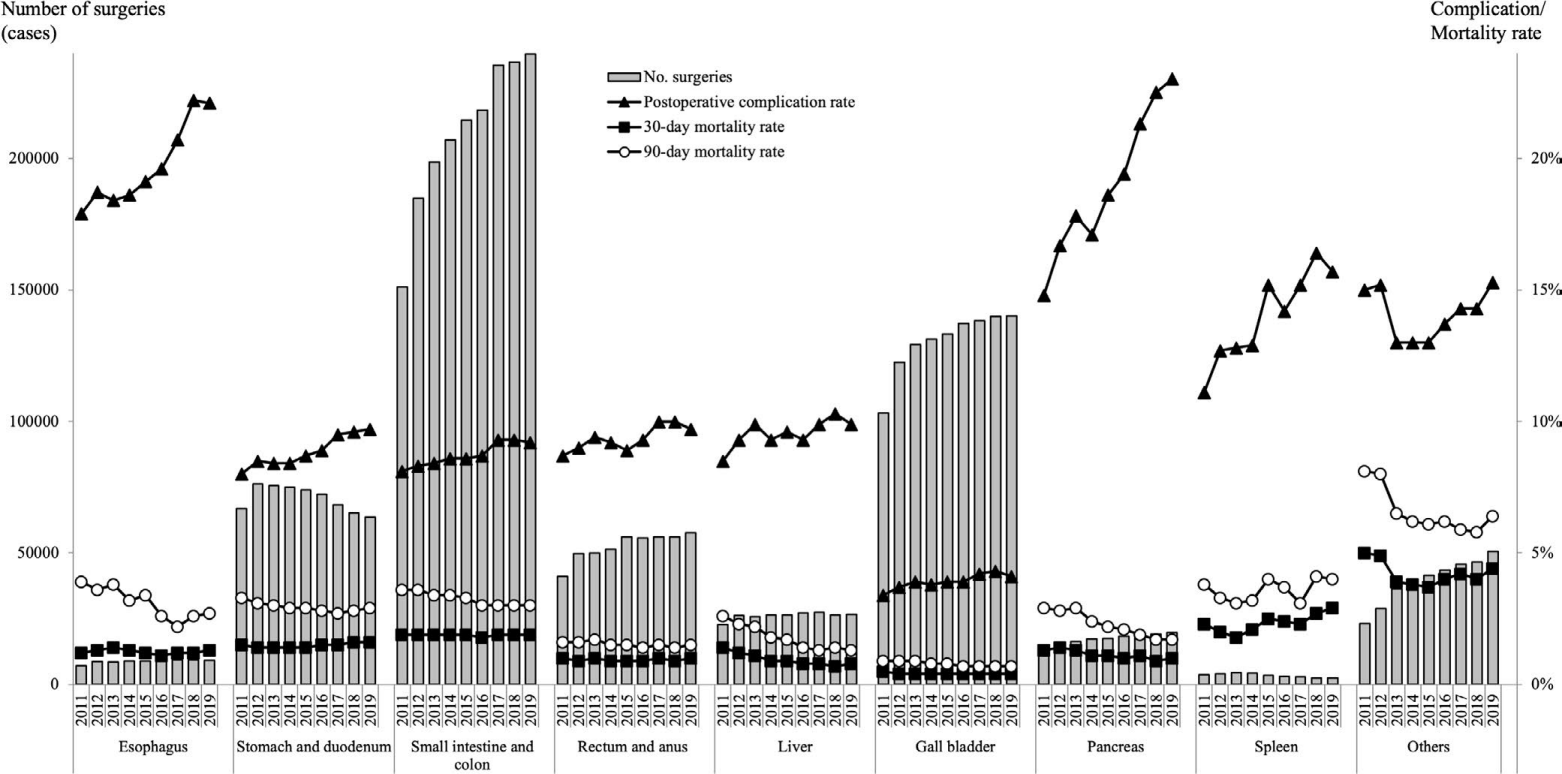
Annual changes in the number of surgeries, 30‐day mortality, operative mortality, and complications: Analysis of the 115 surgical procedures. Postoperative complication rate: the rate of Clavien–Dindo (C–D) classification grade III (complications requiring intervention) or higher complications

The total number of cases represented by the 115 selected gastroenterological surgical procedures, recorded in the NCD from January 1 to December 31, 2019, was 609,589, and 5,029,764 cases were registered between 2011 and 2019 (Figure 1). Regarding organ involvement, the stomach and duodenum decreased slightly to 63,160 (10.3% of all 115 procedures) in 2019 from 65 ,52 (10.8%) in 2018, and the rectum and anus increased slightly to 57,706 (9.5%) in 2019 from 56,162 (9.3%) in 2018. The involvement of other organs (esophagus, small intestine and colon, liver, gallbladder, pancreas, spleen, and other organs) was approximately the same as in 2018 (Table 1).

**TABLE 1:** Annual changes of surgeries by sex, age group, and organ for the selected 115 gastrointestinal operative procedures in the training curriculum for board‐certified surgeons in gastroenterology

Organ	Year	No. surgeries	Percentage by sex		Percentage according to age group (years)					
			Male	Female	<60	60 to <65	65 to <70	70 to <75	75 to <80	≥80
Esophagus	2011	7246	81.8	18.2	22.5	19.6	21.1	18.7	12.0	6.0
	2012	8819	82.2	17.8	22.1	19.7	20.0	19.5	12.9	6.0
	2013	8642	81.5	18.5	20.8	17.5	21.0	20.6	13.2	6.9
	2014	9021	81.5	18.4	20.8	16.5	21.4	20.9	13.8	6.6
	2015	8943	80.8	19.2	19.6	15.3	22.4	22.5	13.1	7.1
	2016	9212	79.6	20.4	20.1	14.4	22.9	20.5	14.5	7.5
	2017	9359	80.0	20.0	19.3	13.4	24.4	19.4	15.5	8.0
	2018	9286	78.4	21.6	19.0	12.8	21.3	21.6	16.7	8.7
	2019	9224	78.6	21.4	18.8	13.1	19.4	22.8	17.3	8.6
Stomach and duodenum	2011	66 740	68.0	32.0	20.1	14.4	14.0	17.1	16.4	18.0
	2012	76 186	68.3	31.7	18.9	14.4	14.5	17.1	16.4	18.6
	2013	75 583	67.9	32.1	18.6	13.1	15.5	17.2	16.9	18.7
	2014	74 920	67.6	32.4	17.9	12.1	16.0	17.8	16.7	19.5
	2015	73 877	67.8	32.2	17.4	11.1	17.1	17.8	16.6	19.9
	2016	72 234	67.8	32.2	17.0	10.2	18.1	17.1	16.6	21.0
	2017	68 287	67.2	32.8	16.3	9.9	17.5	17.3	17.2	21.8
	2018	65 152	66.9	33.1	16.0	9.0	16.4	18.0	17.5	23.2
	2019	63 610	66.5	33.5	15.6	8.8	15.0	19.0	18.5	23.2
Small intestine and colon	2011	151 143	56.7	43.3	37.4	10.9	10.5	12.1	12.2	16.9
	2012	184 810	56.7	43.3	36.4	10.7	10.7	12.2	12.5	17.4
	2013	198 677	56.9	43.1	35.6	10.1	11.3	12.7	12.4	17.8
	2014	206 857	56.9	43.1	34.7	9.4	12.0	13.1	12.4	18.4
	2015	214 453	57.1	42.9	34.0	8.9	12.9	13.1	12.3	18.7
	2016	218 228	57.3	42.7	33.7	8.4	13.6	12.5	12.4	19.3
	2017	235 359	56.7	43.3	32.7	8.0	13.2	12.7	12.9	20.5
	2018	236 496	56.9	43.1	32.2	7.7	12.6	13.4	13.2	21.1
	2019	239 612	56.3	43.7	32.1	7.4	11.7	13.9	13.5	21.2
Rectum and anus	2011	41 061	59.1	40.9	22.0	16.1	14.6	15.4	14.2	17.7
	2012	49 704	58.3	41.7	22.3	14.8	14.6	15.5	14.3	18.5
	2013	49 980	58.0	42.0	20.9	13.9	15.2	16.1	14.6	19.3
	2014	51 454	58.3	41.7	20.4	13.1	16.0	16.4	14.2	19.9
	2015	56 092	57.8	42.2	22.3	11.8	16.7	15.7	14.0	19.4
	2016	55 666	57.3	42.7	22.0	11.1	17.9	15.0	13.6	20.4
	2017	56 144	56.7	43.3	22.2	10.2	17.3	15.1	14.2	21.0
	2018	56 162	56.9	43.1	22.2	9.8	15.9	15.8	14.6	21.6
	2019	57 706	56.3	43.7	22.5	9.5	14.8	16.5	14.9	21.9
Liver	2011	22 855	67.3	32.7	22.2	16.5	16.3	18.7	17.2	9.2
	2012	26 288	66.3	33.7	22.1	15.7	16.7	18.0	17.4	10.2
	2013	25 814	66.1	33.9	21.3	14.6	17.6	18.7	17.3	10.5
	2014	26 518	66.3	33.7	21.5	13.7	18.1	19.8	16.6	10.3
	2015	26 378	65.7	34.3	20.8	12.8	18.9	19.4	16.5	11.5
	2016	27 212	66.4	33.6	20.3	11.5	20.5	18.6	17.0	12.1
	2017	27 397	65.8	34.2	20.1	11.0	20.2	18.8	17.2	12.7
	2018	26 531	66.5	33.5	19.6	10.3	18.8	19.6	17.8	13.8
	2019	26 582	66.3	33.7	19.4	10.1	16.5	21.1	18.6	14.2
Gall bladder	2011	103 183	54.5	45.4	34.3	14.0	12.2	13.8	12.8	13.0
	2012	122 513	55.2	44.8	32.9	13.8	12.4	13.9	13.2	13.8
	2013	129 162	55.3	44.7	32.6	12.9	13.0	14.2	13.2	14.0
	2014	131 182	55.6	44.4	32.1	11.8	13.9	14.5	13.2	14.5
	2015	133 126	55.6	44.4	32.0	11.2	15.0	14.1	13.0	14.8
	2016	137 360	55.4	44.6	32.6	10.6	15.5	13.1	12.9	15.3
	2017	138 267	55.6	44.4	32.2	10.2	15.1	13.5	13.2	15.8
	2018	139 844	55.3	44.7	31.8	9.7	14.2	14.2	13.4	16.7
	2019	140 214	55.4	44.6	31.6	9.6	13.3	14.7	13.9	16.9
Pancreas	2011	13 477	59.9	40.1	20.0	15.6	16.9	19.7	17.7	10.2
	2012	15 550	60.0	40.0	19.8	15.2	17.0	19.5	18.2	10.3
	2013	16 380	59.7	40.3	19.1	13.6	18.0	20.7	17.7	10.9
	2014	17 313	59.5	40.5	18.4	12.4	19.0	21.0	18.2	11.1
	2015	17 407	59.1	40.9	18.2	11.3	19.4	21.6	18.1	11.4
	2016	18 238	58.9	41.1	18.2	10.4	19.9	20.4	19.0	12.2
	2017	19 138	59.2	40.8	17.7	9.9	19.5	19.9	20.1	12.9
	2018	19 152	58.6	41.4	16.9	9.2	18.2	21.5	20.4	13.7
	2019	19 703	58.3	41.7	17.0	9.2	16.5	21.6	21.1	14.6
Spleen	2011	3609	61.3	38.7	35.3	15.6	14.7	14.8	11.9	7.8
	2012	4142	61.4	38.6	32.9	16.3	15.0	15.1	12.9	7.8
	2013	4509	61.8	38.2	30.8	14.9	15.9	16.5	13.1	8.7
	2014	4272	61.8	38.2	29.9	13.0	17.3	17.0	13.8	9.1
	2015	3568	60.4	39.6	29.7	11.4	17.3	16.6	14.1	10.8
	2016	3171	57.3	42.7	31.9	11.7	17.7	15.7	12.5	10.5
	2017	2864	58.7	41.3	31.6	11.0	18.1	16.0	13.3	10.0
	2018	2544	56.6	43.4	32.6	9.9	15.6	16.9	13.9	11.1
	2019	2413	55.2	44.8	31.3	10.5	16.8	15.8	13.1	12.5
Others	2011	23 218	55.0	45.0	32.0	11.9	11.3	13.3	13.8	17.6
	2012	28 779	55.4	44.6	31.1	11.7	11.7	13.8	13.7	18.0
	2013	36 363	53.1	46.9	28.3	10.9	12.7	14.1	14.8	19.1
	2014	39 854	53.7	46.3	28.1	10.1	13.1	14.5	14.4	19.8
	2015	41 465	53.2	46.8	27.4	9.4	14.0	14.5	14.2	20.6
	2016	43 523	54.0	46.0	27.5	9.2	14.6	13.5	14.0	21.2
	2017	45 622	54.1	45.9	27.0	8.2	14.7	13.5	14.6	21.9
	2018	46 587	54.1	45.9	26.8	8.2	14.0	14.4	14.7	21.9
	2019	50 525	54.8	45.2	27.0	8.1	12.7	15.3	15.0	21.9

Most cases were performed in the certified or related institutions of JSGS and included a notably high number of operations involving the esophagus (certified 94.3%, related 3.8%), liver (certified 89.7%, related 6.8%), and pancreas (certified 91.9%, related 6.2%), indicating that very few cases, fewer than 2–4%, underwent these surgeries in nonrelated or noncertified institutions in Japan.

Regarding the surgeons’ credentials, it was clear that an increased number of board‐certified surgeons participated in the surgeries; these were in the esophagus (94.2%), stomach and duodenum (83.8%), small intestine and colon (74.0%), liver (94.1%), gallbladder (75.7%), pancreas (95.1%), spleen (86.8%), and other organs (74.0%) (Table 2). Meanwhile, the rate of surgeries by nonboard‐certified surgeons decreased slightly but remained at more than 50% in the gallbladder (67.7%), small intestine and colon (66.8%), stomach and duodenum (53.9%), and rectum and anus (51.1%), while they were below 35% in the liver (33.6%), pancreas (30.8%), and esophagus (23.6%).

**TABLE 2:** Institution and anesthesiologist and specialist participation rates by organ for the selected 115 gastrointestinal operative procedures

Organ	Year	No. surgeries	Percentage by institution group			Anesthesiologist Prticipation (%)	Board‐certified surgeon participation (%)	Medical practitioners (%)	
			Certified institution	Related institution	Other			Board‐certified Surgeons	Nonboard‐certified surgeons
Esophagus	2011	7246	93.5	5.9	0.6	97.0	87.0	62.8	37.2
	2012	8819	78.1	5.9	16.0	97.4	87.0	62.7	37.3
	2013	8642	90.6	7.1	2.4	97.3	88.4	64.4	35.6
	2014	9021	91.1	6.1	2.8	97.9	90.1	67.6	32.4
	2015	8943	91.5	6.0	2.5	97.9	91.1	69.4	30.6
	2016	9212	92.4	5.0	2.6	98.2	91.2	70.0	30.0
	2017	9359	92.7	4.0	3.3	97.9	92.5	71.8	28.2
	2018	9286	93.8	4.0	2.2	98.5	94.7	75.2	24.8
	2019	9224	94.3	3.8	1.9	98.4	94.2	76.4	23.6
Stomach and duodenum	2011	66 740	80.2	17.3	2.6	92.8	69.3	35.1	64.9
	2012	76 186	63.5	15.6	20.9	93.5	70.3	35.6	64.4
	2013	75 583	76.3	19.3	4.4	93.3	73.5	37.7	62.3
	2014	74 920	77.0	18.2	4.8	93.6	75.9	39.2	60.8
	2015	73 877	77.1	18.3	4.6	93.9	76.1	39.2	60.8
	2016	72 234	79.6	16.1	4.3	94.6	78.7	41.0	59.0
	2017	68 287	79.6	15.3	5.1	94.8	79.7	41.8	58.2
	2018	65 152	80.0	14.8	5.1	95.1	81.4	43.2	56.8
	2019	63 610	81.3	14.2	4.5	95.4	83.8	46.1	53.9
Small intestine and colon	2011	151 143	76.8	20.2	2.9	88.1	59.2	25.1	74.9
	2012	184 810	60.6	18.2	21.2	88.9	59.9	25.4	74.6
	2013	198 677	72.6	22.2	5.2	89.6	62.7	26.6	73.4
	2014	206 857	73.0	21.4	5.6	90.8	65.4	28.1	71.9
	2015	214 453	73.8	20.7	5.5	91.6	66.3	28.5	71.5
	2016	218 228	75.6	19.0	5.5	92.4	68.1	29.5	70.5
	2017	235 359	76.0	18.0	6.0	92.9	70.1	31.1	68.9
	2018	236 496	76.3	17.5	6.1	93.3	71.8	32.6	67.4
	2019	239 612	77.1	17.1	5.8	94.1	74.0	33.2	66.8
Rectum and anus	2011	41 061	76.9	19.0	4.1	86.3	68.3	36.9	63.1
	2012	49 704	60.4	18.2	21.4	85.7	68.6	37.6	62.4
	2013	49 980	72.9	21.7	5.4	87.3	71.2	39.4	60.6
	2014	51 454	73.5	20.9	5.6	87.9	73.7	41.6	58.4
	2015	56 092	72.5	20.8	6.7	84.9	73.5	41.5	58.5
	2016	55 666	74.1	19.4	6.6	85.7	74.7	42.1	57.9
	2017	56 144	73.8	18.2	8.0	84.8	76.1	43.9	56.1
	2018	56 162	74.1	17.9	8.0	85.2	77.2	46.7	53.3
	2019	57 706	74.9	17.3	7.8	86.0	80.1	48.9	51.1
Liver	2011	22 855	89.3	9.7	1.1	95.6	85.2	55.2	44.8
	2012	26 288	74.2	9.2	16.7	95.4	85.7	57.4	42.6
	2013	25 814	86.3	10.7	2.9	96.3	87.5	57.1	42.9
	2014	26 518	86.3	10.0	3.7	96.4	89.0	59.6	40.4
	2015	26 378	87.3	9.5	3.2	96.6	89.1	59.1	40.9
	2016	27 212	88.4	8.8	2.9	96.8	90.0	59.6	40.4
	2017	27 397	89.0	7.8	3.1	97.1	91.8	62.5	37.5
	2018	26 531	89.4	7.1	3.5	97.3	92.8	64.1	35.9
	2019	26 582	89.7	6.8	3.6	97.3	94.1	66.4	33.6
Gall bladder	2011	103 183	73.9	22.5	3.6	91.8	61.9	26.4	73.6
	2012	122 513	57.5	19.6	22.9	92.1	62.8	26.3	73.7
	2013	129 162	69.9	24.1	5.9	92.2	65.4	27.3	72.7
	2014	131 182	70.3	23.3	6.4	92.3	67.4	28.1	71.9
	2015	133 126	70.8	22.8	6.4	92.9	68.4	28.1	71.9
	2016	137 360	72.4	21.3	6.3	93.5	69.4	28.9	71.1
	2017	138 267	72.6	20.1	7.3	93.7	71.4	29.9	70.1
	2018	139 844	72.5	20.1	7.4	94.1	73.1	31.1	68.9
	2019	140 214	73.5	19.4	7.1	94.4	75.7	32.3	67.7
Pancreas	2011	13 477	88.1	10.8	1.2	95.8	85.2	57.7	42.3
	2012	15 550	72.8	8.7	18.5	96.3	86.5	59.9	40.1
	2013	16 380	86.5	11.0	2.4	95.9	87.6	60.2	39.8
	2014	17 313	86.9	9.9	3.3	96.2	89.1	61.3	38.7
	2015	17 407	88.4	9.1	2.4	96.4	90.3	61.6	38.4
	2016	18 238	89.8	8.0	2.3	96.8	91.1	62.4	37.6
	2017	19 138	90.4	7.1	2.5	97.2	92.3	63.9	36.1
	2018	19 152	91.3	6.4	2.3	97.3	93.4	66.5	33.5
	2019	19 703	91.9	6.2	1.9	97.2	95.1	69.2	30.8
Spleen	2011	3609	87.0	11.6	1.4	94.6	75.2	44.9	55.1
	2012	4142	70.5	9.5	20.0	81.7	75.8	44.4	55.6
	2013	4509	83.2	13.8	3.0	95.2	75.4	43.3	56.7
	2014	4272	85.4	11.5	3.1	94.6	77.5	45.2	54.8
	2015	3568	85.6	12.3	2.1	94.8	78.9	45.5	54.5
	2016	3171	86.8	10.1	3.1	95.7	80.5	48.0	52.0
	2017	2864	87.4	9.3	3.3	95.3	82.3	49.1	50.9
	2018	2544	86.9	9.7	3.4	95.3	84.7	49.3	50.7
	2019	2413	88.1	8.7	3.2	96.2	86.8	54.0	46.0
Others	2011	23 218	80.2	17.0	2.8	90.3	60.4	27.2	72.8
	2012	28 779	65.7	15.2	19.1	91.0	61.1	27.6	72.4
	2013	36 363	76.1	19.3	4.6	91.5	63.4	28.5	71.5
	2014	39 854	76.6	18.2	5.1	91.9	64.9	29.7	70.3
	2015	41 465	78.0	17.2	4.8	92.4	65.6	29.4	70.6
	2016	43 523	79.4	15.8	4.8	92.7	67.3	30.3	69.7
	2017	45 622	80.1	14.8	5.1	93.1	69.7	32.3	67.7
	2018	46 587	80.2	14.2	5.7	93.8	71.2	33.1	66.9
	2019	50 525	80.9	13.9	5.3	94.3	74.0	35.2	64.8

The rate of postoperative complications and 30‐ and 90‐day mortalities are described in Table 3. The rate of complications in the esophagus, stomach and duodenum, pancreas, and spleen increased slightly toward 2019, while the mortality decreased in these organ groups. The rate of complications and 30‐ 90‐day mortality in the rest of the organs were approximately the same as before.

**TABLE 3:** Number of surgeries and mortality rates according to organ treated using the selected 115 gastrointestinal operative procedures

Organ	Year	No. surgeries	Number of postoperative complications^a^/rate (%)	Number of postoperative 30‐day mortalities/rate (%)	Number of postoperative 90‐day mortalities/rate (%)
Esophagus	2011	7246	1294/17.9	87/1.2	279/3.9
	2012	8819	1653/18.7	117/1.3	315/3.6
	2013	8642	1593/18.4	121/1.4	327/3.8
	2014	9021	1679/18.6	115/1.3	289/3.2
	2015	8943	1709/19.1	103/1.2	304/3.4
	2016	9212	1805/19.6	100/1.1	238/2.6
	2017	9359	1938/20.7	108/1.2	208/2.2
	2018	9286	2065/22.2	108/1.2	246/2.6
	2019	9224	2035/22.1	119/1.3	246/2.7
Stomach and duodenum	2011	66 740	5354/8.0	992/1.5	2183/3.3
	2012	76 186	6447/8.5	1085/1.4	2381/3.1
	2013	75 583	6380/8.4	1059/1.4	2269/3.0
	2014	74 920	6328/8.4	1064/1.4	2174/2.9
	2015	73 877	6418/8.7	1007/1.4	2110/2.9
	2016	72 234	6413/8.9	1066/1.5	2016/2.8
	2017	68 287	6455/9.5	1046/1.5	1863/2.7
	2018	65 152	6228/9.6	1048/1.6	1833/2.8
	2019	63 610	6159/9.7	1022/1.6	1826/2.9
Small intestine and colon	2011	151 143	12184/8.1	2943/1.9	5390/3.6
	2012	184 810	15395/8.3	3564/1.9	6583/3.6
	2013	198 677	16709/8.4	3723/1.9	6803/3.4
	2014	206 857	17776/8.6	3822/1.9	6961/3.4
	2015	214 453	18372/8.6	4019/1.9	7092/3.3
	2016	218 228	19020/8.7	3933/1.8	6621/3.0
	2017	235 359	21854/9.3	4588/1.9	7118/3.0
	2018	236 496	21881/9.3	4452/1.9	7116/3.0
	2019	239 612	22061/9.2	4671/1.9	7298/3.0
Rectum and anus	2011	41 061	3584/8.7	395/1.0	676/1.6
	2012	49 704	4488/9.0	462/0.9	802/1.6
	2013	49 980	4684/9.4	517/1.0	858/1.7
	2014	51 454	4711/9.2	449/0.9	792/1.5
	2015	56 092	4986/8.9	519/0.9	824/1.5
	2016	55 666	5194/9.3	503/0.9	766/1.4
	2017	56 144	5600/10.0	556/1.0	829/1.5
	2018	56 162	5622/10.0	522/0.9	803/1.4
	2019	57 706	5573/9.7	563/1.0	839/1.5
Liver	2011	22 855	1933/8.5	309/1.4	590/2.6
	2012	26 288	2454/9.3	310/1.2	605/2.3
	2013	25 814	2549/9.9	275/1.1	575/2.2
	2014	26 518	2466/9.3	246/0.9	481/1.8
	2015	26 378	2537/9.6	234/0.9	451/1.7
	2016	27 212	2543/9.3	222/0.8	382/1.4
	2017	27 397	2724/9.9	214/0.8	364/1.3
	2018	26 531	2737/10.3	189/0.7	372/1.4
	2019	26 582	2624/9.9	201/0.8	334/1.3
Gall bladder	2011	103 183	3473/3.4	483/0.5	946/0.9
	2012	122 513	4587/3.7	531/0.4	1082/0.9
	2013	129 162	4982/3.9	546/0.4	1130/0.9
	2014	131 182	5020/3.8	569/0.4	1097/0.8
	2015	133 126	5231/3.9	541/0.4	1036/0.8
	2016	137 360	5320/3.9	559/0.4	980/0.7
	2017	138 267	5761/4.2	576/0.4	968/0.7
	2018	139 844	5964/4.3	584/0.4	954/0.7
	2019	140 214	5748/4.1	565/0.4	935/0.7
Pancreas	2011	13 477	1994/14.8	175/1.3	386/2.9
	2012	15 550	2595/16.7	213/1.4	437/2.8
	2013	16 380	2917/17.8	211/1.3	482/2.9
	2014	17 313	2966/17.1	195/1.1	423/2.4
	2015	17 407	3229/18.6	185/1.1	379/2.2
	2016	18 238	3543/19.4	185/1.0	390/2.1
	2017	19 138	4076/21.3	219/1.1	365/1.9
	2018	19 152	4309/22.5	178/0.9	325/1.7
	2019	19 703	4522/23.0	199/1.0	335/1.7
Spleen	2011	3609	400/11.1	83/2.3	137/3.8
	2012	4142	528/12.7	84/2.0	138/3.3
	2013	4509	575/12.8	79/1.8	139/3.1
	2014	4272	549/12.9	88/2.1	137/3.2
	2015	3568	543/15.2	88/2.5	144/4.0
	2016	3171	449/14.2	76/2.4	117/3.7
	2017	2864	434/15.2	65/2.3	89/3.1
	2018	2544	418/16.4	69/2.7	104/4.1
	2019	2413	380/15.7	71/2.9	97/4.0
Others	2011	23 218	3494/15.0	1163/5.0	1887/8.1
	2012	28 779	4388/15.2	1399/4.9	2293/8.0
	2013	36 363	4712/13.0	1401/3.9	2346/6.5
	2014	39 854	5176/13.0	1521/3.8	2489/6.2
	2015	41 465	5380/13.0	1541/3.7	2545/6.1
	2016	43 523	5975/13.7	1760/4.0	2684/6.2
	2017	45 622	6539/14.3	1909/4.2	2699/5.9
	2018	46 587	6645/14.3	1865/4.0	2710/5.8
	2019	50 525	7750/15.3	2221/4.4	3220/6.4

^a^ Complications were defined by Clavien–Dindo grade Ⅲa–Ⅴ.

Among the procedures performed in over 50 cases in 2019 in 115 gastroenterological procedures, those with the highest 90‐day mortality rate were 1) acute pancreatitis surgery (23.2%), 2) esophagus bypass (13.5%), 3) esophageal fistula construction (12.8%), 4) gastrointestinal perforation surgery (11.9%), 5) ADP surgery (11.4%), 6) total colectomy (11.7%), 7) gastric fistula construction (excluding PEG) (11.6%), 8) external cholecystectomy (10.6%), and 9) hepatorrhaphy (10.0%). Among these nine procedures, the degree of difficulty was high in one procedure (esophagus bypass) and either moderate or low in the other eight Tables S1‐1–9).

### 3.2 Eight major operative procedures

**FIGURE 2:**
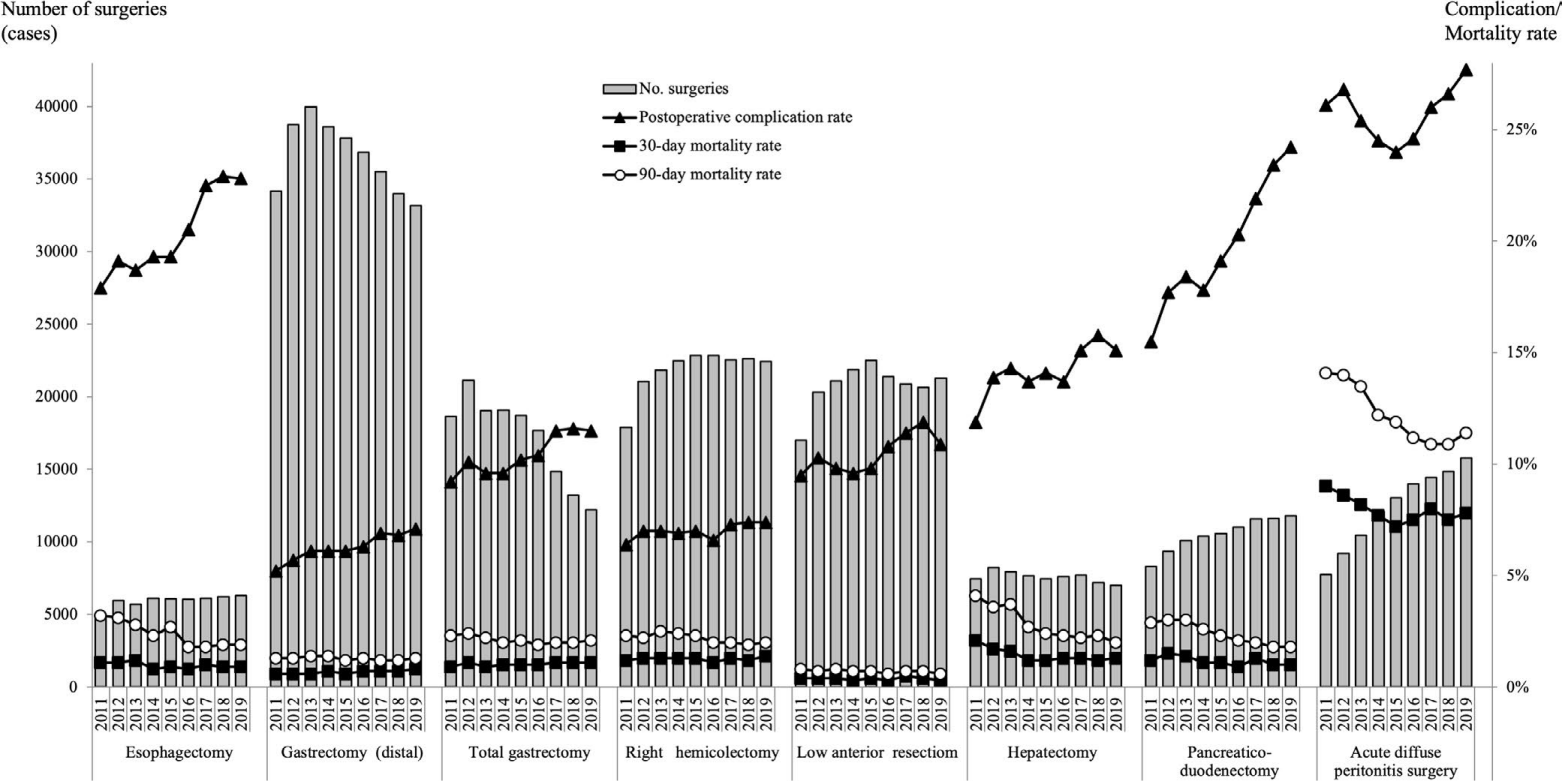
Annual changes in the number of surgeries, 30‐day mortality, operative mortality, and complications: Analysis of the eight major surgical procedures. Postoperative complication rate: the rate of Clavien–Dindo (C–D) classification grade III or higher complications

The number of surgeries carried out annually for the eight major operative procedures, the percentage by gender, and the percentage according to age group between 2011 and 2019 are shown in Table 4 (Figure 2).

**TABLE 4:** Annual changes of surgeries by sex, age group, and organ for eight main operative procedures

Procedure	Year	No. surgeries	Percentage by sex		Percentage according to age group (years)					
			Male	Female	<60	60 to <65	65 to <70	70 to <75	75 to <80	≥80
Esophagectomy	2011	4916	84.1	15.9	20.4	20.8	22.5	19.4	12.2	4.7
	2012	5946	84.4	15.6	19.7	21.3	20.7	20.3	13.1	4.9
	2013	5694	83.6	16.4	18.3	18.3	22.6	21.3	13.8	5.8
	2014	6091	84.0	16.0	18.7	17.8	22.8	22.0	13.4	5.2
	2015	6060	82.9	17.1	17.9	16.3	23.6	23.5	13.1	5.7
	2016	6041	81.7	18.3	17.8	15.8	25.3	21.6	14.3	5.2
	2017	6100	82.3	17.7	17.0	14.6	25.6	20.6	15.8	6.3
	2018	6207	80.5	19.5	17.2	14.2	22.6	22.8	16.8	6.5
	2019	6298	81.0	19.0	17.0	13.9	20.7	24.1	17.2	7.0
Gastrectomy (distal)	2011	34 160	66.6	33.4	18.1	15.0	14.2	17.4	16.8	18.5
	2012	38 750	66.9	33.1	16.9	14.8	15.0	17.8	16.5	18.8
	2013	39 957	66.7	33.3	16.3	13.5	15.8	17.8	17.6	19.0
	2014	38 584	66.4	33.6	15.7	12.4	16.6	18.4	17.3	19.5
	2015	37 819	66.6	33.4	14.8	11.3	17.5	18.2	17.5	20.6
	2016	36 852	66.6	33.4	14.5	10.4	18.5	17.6	17.4	21.6
	2017	35 517	66.8	33.2	13.4	9.9	18.0	18.1	18.0	22.6
	2018	33 988	66.6	33.4	12.9	9.1	16.9	19.0	18.1	24.0
	2019	33 177	66.5	33.5	12.2	8.6	15.3	20.4	19.3	24.3
Total gastrectomy	2011	18 652	73.7	26.3	16.6	14.7	16.0	19.7	18.0	15.0
	2012	21 122	74.2	25.8	15.5	14.8	15.7	19.2	18.5	16.3
	2013	19 035	74.0	26.0	14.7	13.5	16.9	19.4	19.2	16.3
	2014	19 071	73.7	26.3	14.0	12.3	17.2	20.1	18.9	17.5
	2015	18 695	74.5	25.5	13.7	11.1	18.9	20.8	18.2	17.4
	2016	17 670	74.4	25.6	12.6	10.3	19.6	19.5	19.0	19.0
	2017	14 840	74.2	25.8	12.2	9.9	19.0	19.6	19.8	19.5
	2018	13 223	74.4	25.6	10.8	9.1	18.0	20.6	20.6	20.9
	2019	12 188	74.3	25.7	10.7	9.0	16.9	21.4	21.5	20.6
Right hemicolectomy	2011	17 890	50.5	49.5	12.8	11.6	13.1	17.3	18.8	26.5
	2012	21 034	50.3	49.7	13.1	10.9	13.1	17.0	19.0	26.9
	2013	21 814	50.6	49.4	13.0	10.0	13.4	17.6	18.9	27.1
	2014	22 446	50.6	49.4	12.0	9.2	13.8	18.2	18.6	28.2
	2015	22 850	50.5	49.5	11.5	8.6	14.6	18.1	18.1	29.1
	2016	22 829	51.3	48.7	11.4	7.7	15.9	16.7	18.5	29.8
	2017	22 543	50.9	49.1	11.3	7.4	14.9	16.3	19.3	30.8
	2018	22 610	51.4	48.6	10.7	6.9	13.9	17.7	19.6	31.2
	2019	22 410	51.5	48.5	11.0	6.6	12.9	17.7	19.7	32.1
Low anterior resection	2011	16 984	64.8	35.2	24.1	18.5	16.5	16.2	12.9	11.7
	2012	20 321	64.8	35.2	24.2	17.6	16.5	16.6	13.1	12.0
	2013	21 096	64.2	35.8	23.8	16.5	17.4	16.9	13.5	11.8
	2014	21 861	64.8	35.2	23.1	15.7	18.3	17.9	13.1	11.9
	2015	22 493	64.4	35.6	23.5	14.2	19.6	17.1	13.6	12.0
	2016	21 387	64.4	35.6	23.4	13.6	20.7	16.8	13.2	12.2
	2017	20 879	64.2	35.8	23.2	12.6	20.9	16.7	13.5	13.2
	2018	20 636	64.9	35.1	22.9	12.5	19.3	18.0	14.4	12.9
	2019	21 262	63.9	36.1	23.3	11.6	18.4	18.6	14.6	13.5
Hepatectomy (nonlateral segments)	2011	7434	70.4	29.6	20.1	16.4	16.5	20.4	18.0	8.7
	2012	8239	69.5	30.5	19.8	16.1	17.4	19.5	18.5	8.8
	2013	7937	69.4	30.6	19.4	14.2	18.0	20.3	18.2	9.9
	2014	7666	69.2	30.8	18.5	13.8	18.5	21.5	17.6	10.0
	2015	7439	68.9	31.1	18.7	12.5	19.3	20.9	17.6	11.1
	2016	7610	68.7	31.3	18.0	11.9	21.1	20.4	17.5	11.1
	2017	7698	69.5	30.5	17.2	11.3	20.5	20.4	18.7	11.9
	2018	7192	69.5	30.5	17.2	9.6	19.1	21.4	19.4	13.3
	2019	7018	69.2	30.8	16.7	9.2	16.8	22.6	20.9	13.8
Pancreaticoduodenectomy	2011	8305	61.9	38.1	16.1	16.0	17.3	20.9	18.8	10.9
	2012	9329	62.0	38.0	14.7	15.8	18.0	20.6	20.2	10.6
	2013	10 068	60.9	39.1	14.0	12.6	19.6	22.5	19.4	11.8
	2014	10 400	59.5	40.5	18.4	12.4	19.0	21.0	18.2	11.1
	2015	10 576	60.7	39.3	14.2	11.7	20.0	22.9	19.3	12.0
	2016	11 028	61.1	38.9	14.2	10.3	20.6	21.8	20.3	12.7
	2017	11 580	61.1	38.9	13.8	9.8	20.4	20.8	21.6	13.6
	2018	11 626	60.3	39.7	13.3	9.1	18.9	22.2	22.0	14.6
	2019	11 813	60.7	39.3	13.1	9.1	17.4	22.6	22.1	15.6
Acute diffuse peritonitis surgery	2011	7753	60.0	40.0	31.4	11.2	9.7	11.7	13.2	22.9
	2012	9177	61.0	39.0	30.3	11.2	10.1	11.6	13.4	23.4
	2013	10 447	60.1	39.9	29.1	10.3	11.5	11.8	13.1	24.1
	2014	12 085	61.2	38.8	28.4	9.5	12.2	12.3	12.9	24.7
	2015	13 030	59.4	40.6	28.2	8.9	12.5	13.1	12.3	25.0
	2016	13 981	60.2	39.8	27.4	8.6	13.4	12.4	12.3	26.0
	2017	14 423	59.4	40.6	26.5	7.8	13.0	12.0	13.6	27.1
	2018	14 835	59.2	40.8	26.1	7.7	12.7	13.1	13.5	26.9
	2019	15 765	59.2	40.8	25.2	7.7	11.6	13.6	14.1	27.7

The number of procedures increased in RHC, LAR, and PD and decreased in DG, TG, and HEP.

The age distributions mirrored the tendency in recent years to shift toward older patients, and the percentage of cases with the age >70 years in 2019 (increase from 2011) was 48.3% (+12.0%), 64.0% (+12.3%), 63.5% (+10.8%), 69.5% (+7.1%), 46.7% (+5.9%), 57.3% (+10.2%), 60.3% (+9.7%), 55.4% (+7.6%) in ESO, DG, TG, RHC, LAR, HEP, PD, and ADP, respectively.

The rate of involvement of board‐certified surgeons for the 115 procedures remained the same as in 2018, and the rates have been continuously increasing in recent years; these were approximately as follows: ESO (96.4%), HEP (96.3%), and PD (95.5%), and the lower rates ranged from 73.3–86.8% in DG/TG, RHC, LAR, and ADP (Table 5).

**TABLE 5:** Institution and anesthesiologist and specialist participation rates by organ for eight main operative procedures

Procedure	Year	No. surgeries	Percentage by institution group			Anesthesiologist participation (%)	Board‐certified Surgeon participation (%)	Medical practitioners (%)	
			Certified institution	Related institution	Other			Board‐certified Surgeons	Nonboard‐certified surgeons
Esophagectomy	2011	4916	94.2	5.3	0.5	97.6	88.4	63.5	36.5
	2012	5946	78.3	4.9	16.8	98.1	89.0	64.8	35.2
	2013	5694	92.9	5.9	1.2	98.0	90.8	66.6	33.4
	2014	6091	93.6	4.7	1.7	98.6	92.6	70.2	29.8
	2015	6060	93.6	4.6	1.8	98.5	93.5	72.1	27.9
	2016	6041	94.5	3.8	1.7	98.8	93.7	73.2	26.8
	2017	6100	95.3	3.1	1.7	98.8	94.8	74.7	25.3
	2018	6207	95.9	2.7	1.4	99.1	96.6	78.8	21.2
	2019	6298	96.3	2.3	1.5	98.9	96.4	80.6	19.4
Gastrectomy (distal)	2011	34 160	81.1	16.6	2.3	93.2	71.3	37.0	63.0
	2012	38 750	64.5	15.2	20.3	93.9	72.5	37.9	62.1
	2013	39 957	76.6	19.2	4.1	93.6	76.1	40.6	59.4
	2014	38 584	77.7	17.8	4.5	94.0	78.4	42.1	57.9
	2015	37 819	77.3	18.3	4.4	94.1	78.1	41.3	58.7
	2016	36 852	80.2	15.9	4.0	95.0	81.8	43.8	56.2
	2017	35 517	80.2	14.9	4.8	95.4	82.4	45.2	54.8
	2018	33 988	80.7	14.4	4.8	95.6	84.2	46.6	53.4
	2019	33 177	82.4	13.5	4.0	95.7	86.4	50.1	49.9
Total gastrectomy	2011	18 652	80.9	16.8	2.3	93.9	71.6	37.4	62.6
	2012	21 122	63.0	15.3	21.7	94.3	72.1	38.0	62.0
	2013	19 035	77.2	18.9	3.9	94.2	75.0	39.5	60.5
	2014	19 071	77.8	17.9	4.3	94.4	77.7	41.7	58.3
	2015	18 695	77.9	17.9	4.1	94.5	78.2	42.6	57.4
	2016	17 670	80.0	15.9	4.0	95.0	81.4	45.0	55.0
	2017	14 840	79.3	15.8	4.9	95.0	80.7	44.3	55.7
	2018	13 223	79.6	15.5	4.9	95.4	82.6	46.2	53.8
	2019	12 188	80.0	15.5	4.4	95.7	85.5	49.2	50.8
Right hemicolectomy	2011	17 890	75.7	21.2	3.1	92.7	66.0	30.5	69.5
	2012	21 034	60.0	18.3	21.7	93.0	67.1	30.8	69.2
	2013	21 814	72.1	22.3	5.6	92.9	69.7	32.6	67.4
	2014	22 446	71.2	23.1	5.7	93.4	71.9	33.6	66.4
	2015	22 850	72.1	22.0	5.9	94.1	72.4	33.5	66.5
	2016	22 829	73.8	20.1	6.1	94.5	74.2	34.3	65.7
	2017	22 543	75.0	18.4	6.6	94.7	76.4	37.1	62.9
	2018	22 610	74.8	19.0	6.2	94.7	77.8	38.2	61.8
	2019	22 410	75.8	18.1	6.1	95.6	80.1	39.2	60.8
Low anterior resection	2011	16 984	79.4	17.7	2.9	93.4	72.7	41.6	58.4
	2012	20 321	64.0	16.2	19.7	93.8	73.0	42.3	57.7
	2013	21 096	76.3	19.5	4.2	93.7	75.5	44.3	55.7
	2014	21 861	76.2	19.0	4.9	94.4	78.2	47.2	52.8
	2015	22 493	76.9	18.3	4.8	94.6	79.2	47.7	52.3
	2016	21 387	79.0	16.4	4.7	95.0	81.0	48.8	51.2
	2017	20 879	79.3	15.6	5.1	95.2	83.1	51.2	48.8
	2018	20 636	80.9	14.3	4.8	95.2	84.5	54.4	45.6
	2019	21 262	81.2	14.1	4.6	95.6	86.8	58.3	41.7
Hepatectomy (nonlateral segments)	2011	7434	91.1	8.0	0.8	96.4	88.9	61.5	38.5
	2012	8239	75.9	7.9	16.3	96.8	89.3	64.0	36.0
	2013	7937	88.1	9.7	2.2	96.9	91.0	65.2	34.8
	2014	7666	88.2	8.7	3.1	96.7	92.3	66.6	33.4
	2015	7439	89.2	8.6	2.2	97.2	92.3	66.6	33.4
	2016	7610	90.7	7.1	2.1	97.1	93.3	67.7	32.3
	2017	7698	91.2	6.6	2.2	97.7	95.1	72.3	27.7
	2018	7192	92.8	5.2	2.0	97.7	95.8	72.8	27.2
	2019	7018	92.7	5.2	2.1	97.8	96.3	74.2	25.8
Pancreaticoduodenectomy	2011	8305	87.8	11.0	1.2	95.9	85.7	58.7	41.3
	2012	9329	72.4	8.8	18.8	96.6	87.2	60.9	39.1
	2013	10 068	85.9	11.7	2.4	96.0	87.9	60.5	39.5
	2014	10 400	86.4	10.4	3.3	96.4	90.3	62.2	37.8
	2015	10 576	88.5	9.2	2.4	96.9	90.9	62.1	37.9
	2016	11 028	89.4	8.3	2.3	97.1	91.7	63.3	36.7
	2017	11 580	90.5	7.2	2.3	97.3	93.0	65.0	35.0
	2018	11 626	91.4	6.4	2.2	97.4	94.0	67.6	32.4
	2019	11 813	92.0	6.2	1.9	97.2	95.5	69.6	30.4
Acute diffuse peritonitis surgery	2011	7753	80.6	16.9	2.4	90.0	58.5	23.5	76.5
	2012	9177	65.2	16.4	18.4	90.4	59.4	22.7	77.3
	2013	10 447	77.7	18.1	4.2	91.2	62.4	23.9	76.1
	2014	12 085	77.7	17.2	5.1	91.9	63.3	25.1	74.9
	2015	13 030	79.8	15.9	4.3	92.2	64.5	24.9	75.1
	2016	13 981	82.2	13.8	4.0	93.0	66.8	26.1	73.9
	2017	14 423	83.1	13.0	3.8	93.3	69.0	27.2	72.8
	2018	14 835	83.4	12.4	4.2	93.6	70.4	28.7	71.3
	2019	15 765	83.8	12.2	4.0	94.6	73.7	29.8	70.2

The morbidities and mortalities of these eight major gastroenterological procedures remained relatively constant in all procedures. The morbidities increased in ESO, HEP, and PD compared to those in 2016 or 2017; in contrast, the mortalities decreased in these procedures. In the other five procedures, the rate of morbidities and mortalities remained approximately the same from 2011 to 2019 (Table 6).

**TABLE 6:** Number of surgeries and mortality rates according to organ treated using the eight main operative procedures

Procedure	Year	No. surgeries	No. postoperative complications^a^/rate (%)	No. re‐operation/rate (%)	No. postoperative	No. postoperative
					30‐day mortalities/rate (%)	90‐day mortalities/rate (%)
Esophagectomy	2011	4916	879/17.9	310/6.3	55/1.1	158/3.2
	2012	5946	1135/19.1	345/5.8	63/1.1	183/3.1
	2013	5694	1067/18.7	375/6.6	67/1.2	161/2.8
	2014	6091	1178/19.3	367/6.0	49/0.8	140/2.3
	2015	6060	1171/19.3	392/6.5	57/0.9	166/2.7
	2016	6041	1240/20.5	357/5.9	49/0.8	109/1.8
	2017	6100	1374/22.5	355/5.8	61/1.0	108/1.8
	2018	6207	1420/22.9	367/5.9	53/0.9	115/1.9
	2019	6298	1435/22.8	353/5.6	54/0.9	120/1.9
Gastrectomy (distal)	2011	34 160	1774/5.2	709/2.1	208/0.6	451/1.3
	2012	38 750	2205/5.7	849/2.2	232/0.6	516/1.3
	2013	39 957	2450/6.1	892/2.2	239/0.6	542/1.4
	2014	38 584	2356/6.1	941/2.4	264/0.7	523/1.4
	2015	37 819	2325/6.1	851/2.3	222/0.6	452/1.2
	2016	36 852	2314/6.3	825/2.2	249/0.7	473/1.3
	2017	35 517	2445/6.9	859/2.4	253/0.7	437/1.2
	2018	33 988	2327/6.8	737/2.2	227/0.7	393/1.2
	2019	33 177	2361/7.1	739/2.2	253/0.8	427/1.3
Total gastrectomy	2011	18 652	1716/9.2	634/3.4	177/0.9	427/2.3
	2012	21 122	2135/10.1	758/3.6	224/1.1	503/2.4
	2013	19 035	1831/9.6	642/3.4	169/0.9	428/2.2
	2014	19 071	1840/9.6	698/3.7	185/1.0	379/2.0
	2015	18 695	1907/10.2	654/3.5	178/1.0	387/2.1
	2016	17 670	1835/10.4	638/3.6	174/1.0	336/1.9
	2017	14 840	1702/11.5	514/3.5	161/1.1	293/2.0
	2018	13 223	1529/11.6	487/3.7	148/1.1	265/2.0
	2019	12 188	1406/11.5	427/3.5	136/1.1	258/2.1
Right hemicolectomy	2011	17 890	1150/6.4	588/3.3	213/1.2	410/2.3
	2012	21 034	1470/7.0	677/3.2	263/1.3	471/2.2
	2013	21 814	1527/7.0	721/3.3	280/1.3	538/2.5
	2014	22 446	1544/6.9	771/3.4	287/1.3	530/2.4
	2015	22 850	1607/7.0	769/3.4	301/1.3	534/2.3
	2016	22 829	1510/6.6	791/3.5	253/1.1	449/2.0
	2017	22 543	1648/7.3	785/3.5	296/1.3	450/2.0
	2018	22 610	1679/7.4	740/3.3	276/1.2	424/1.9
	2019	22 410	1666/7.4	713/3.2	306/1.4	449/2.0
Low anterior resection	2011	16 984	1616/9.5	1213/7.1	75/0.4	136/0.8
	2012	20 321	2092/10.3	1413/6.9	88/0.4	149/0.7
	2013	21 096	2059/9.8	1473/7.0	80/0.4	175/0.8
	2014	21 861	2098/9.6	1546/7.1	70/0.3	152/0.7
	2015	22 493	2210/9.8	1550/6.9	95/0.4	156/0.7
	2016	21 387	2306/10.8	1492/7.0	68/0.3	126/0.6
	2017	20 879	2376/11.4	1330/6.4	96/0.5	148/0.7
	2018	20 636	2454/11.9	1424/6.9	90/0.4	142/0.7
	2019	21 262	2320/10.9	1346/6.3	73/0.3	119/0.6
Hepatectomy (nonlateral segments)	2011	7434	886/11.9	203/2.7	155/2.1	303/4.1
	2012	8239	1146/13.9	248/3.0	142/1.7	293/3.6
	2013	7937	1135/14.3	226/2.8	130/1.6	290/3.7
	2014	7666	1052/13.7	242/3.2	94/1.2	208/2.7
	2015	7439	1049/14.1	213/2.9	87/1.2	182/2.4
	2016	7610	1046/13.7	220/2.9	96/1.3	178/2.3
	2017	7698	1160/15.1	221/2.9	97/1.3	169/2.2
	2018	7192	1137/15.8	211/2.9	83/1.2	163/2.3
	2019	7018	1058/15.1	189/2.7	94/1.3	143/2.0
Pancreaticoduodenectomy	2011	8305	1285/15.5	299/3.6	97/1.2	238/2.9
	2012	9329	1654/17.7	365/3.9	137/1.5	281/3.0
	2013	10 068	1853/18.4	407/4.0	142/1.4	307/3.0
	2014	10 400	1847/17.8	374/3.6	111/1.1	267/2.6
	2015	10 576	2025/19.1	378/3.6	120/1.1	247/2.3
	2016	11 028	2242/20.3	393/3.6	98/0.9	232/2.1
	2017	11 580	2539/21.9	413/3.6	145/1.3	232/2.0
	2018	11 626	2716/23.4	402/3.5	111/1.0	204/1.8
	2019	11 813	2854/24.2	402/3.4	119/1.0	210/1.8
Acute diffuse peritonitis surgery	2011	7753	2022/26.1	634/8.2	697/9.0	1096/14.1
	2012	9177	2456/26.8	685/7.5	785/8.6	1289/14.0
	2013	10 447	2652/25.4	786/7.5	861/8.2	1408/13.5
	2014	12 085	2966/24.5	937/7.8	927/7.7	1472/12.2
	2015	13 030	3126/24.0	1051/8.1	943/7.2	1551/11.9
	2016	13 981	3445/24.6	1068/7.6	1052/7.5	1572/11.2
	2017	14 423	3756/26.0	1125/7.8	1152/8.0	1575/10.9
	2018	14 835	3943/26.6	1183/8.0	1117/7.5	1617/10.9
	2019	15 765	4367/27.7	1247/7.9	1233/7.8	1795/11.4

^a^ Complications were defined by Clavien–Dindo grade IIIa–V.

**FIGURE 3:**
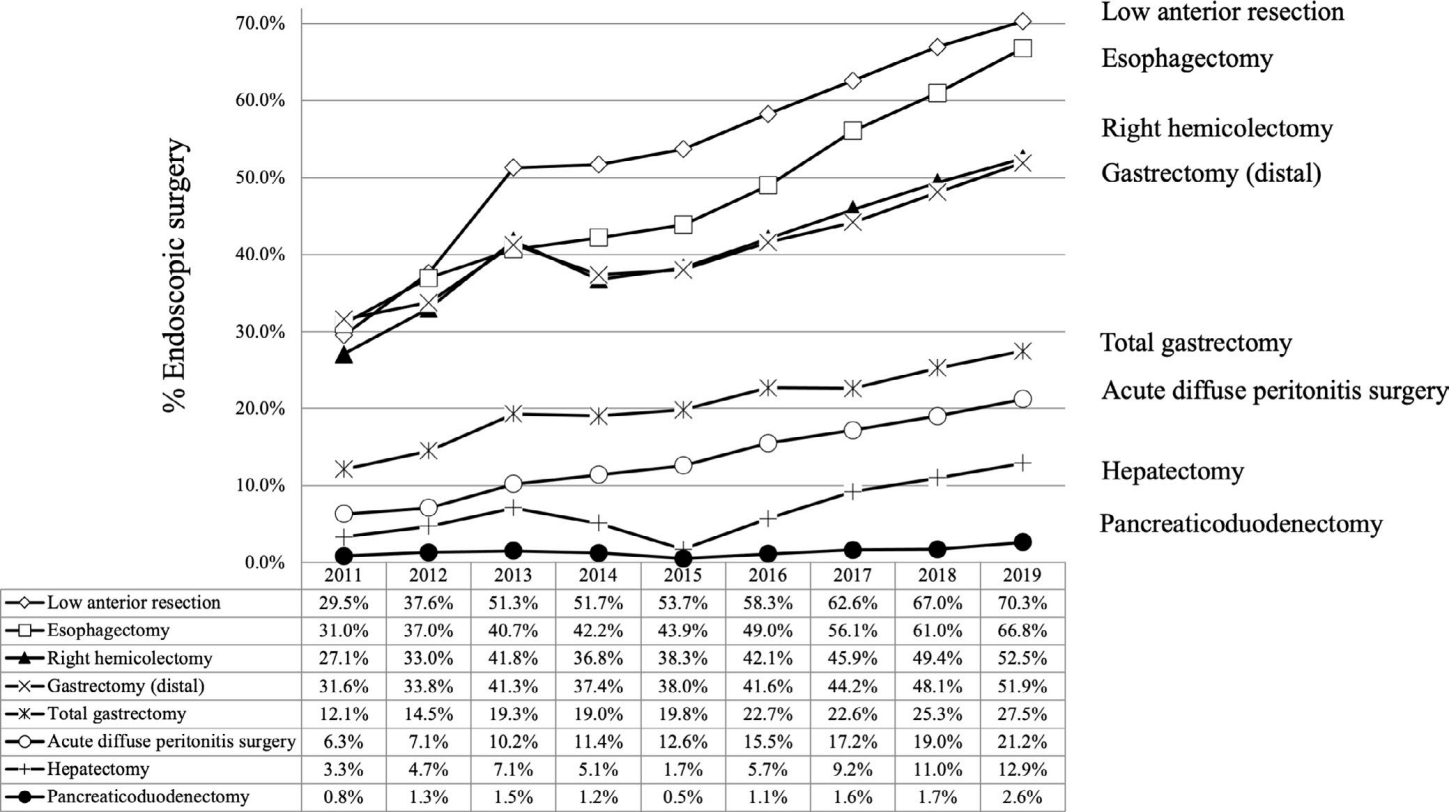
Annual changes in the percentage of surgeries performed endoscopically: analysis of the eight major surgical procedures

The rate of laparoscopic surgery in 2019 continuously increased from 2011 in LAR (70.3%, +40.8%), ESO (66.8%, +34.8%), RHC (52.5%, +25.4%), DG (51.9%, +20.3%), TG (27.5%, +15.4%), HEP (nonlateral segment) (12.9%, +9.6%), and ADP (21.2%, +14.9%), with the exception of PD, which remained low at 2.6% (+1.8%) (Table 7, Figure 3).

**TABLE 7:** Annual changes of endoscopic surgeries for eight main operative procedures

Procedure	Year	No. surgeries	Endoscopic surgery	%Endoscopic surgery
Esophagectomy	2011	4916	1525	31.0
	2012	5946	2200	37.0
	2013	5694	2315	40.7
	2014	6091	2569	42.2
	2015	6060	2659	43.9
	2016	6041	2961	49.0
	2017	6100	3424	56.1
	2018	6207	3788	61.0
	2019	6298	4209	66.8
Gastrectomy (distal)	2011	34 160	10 801	31.6
	2012	38 750	13 098	33.8
	2013	39 957	16 507	41.3
	2014	38 584	14 432	37.4
	2015	37 819	14 357	38.0
	2016	36 852	15 333	41.6
	2017	35 517	15 696	44.2
	2018	33 988	16 355	48.1
	2019	33 177	17 205	51.9
Total gastrectomy	2011	18 652	2258	12.1
	2012	21 122	3060	14.5
	2013	19 035	3669	19.3
	2014	19 071	3620	19.0
	2015	18 695	3707	19.8
	2016	17 670	4007	22.7
	2017	14 840	3347	22.6
	2018	13 223	3344	25.3
	2019	12 188	3351	27.5
Right hemicolectomy	2011	17 890	4842	27.1
	2012	21 034	6954	33.0
	2013	21 814	9124	41.8
	2014	22 446	8269	36.8
	2015	22 850	8755	38.3
	2016	22 829	9622	42.1
	2017	22 543	10 341	45.9
	2018	22 610	11 165	49.4
	2019	22 410	11 769	52.5
Low anterior resection	2011	16 984	5018	29.5
	2012	20 321	7649	37.6
	2013	21 096	10 814	51.3
	2014	21 861	11 298	51.7
	2015	22 493	12 080	53.7
	2016	21 387	12 478	58.3
	2017	20 879	13 064	62.6
	2018	20 636	13 825	67.0
	2019	21 262	14 950	70.3
Hepatectomy (nonlateral segments)	2011	7434	242	3.3
	2012	8239	389	4.7
	2013	7937	567	7.1
	2014	7666	392	5.1
	2015	7439	127	1.7
	2016	7610	433	5.7
	2017	7698	712	9.2
	2018	7192	791	11.0
	2019	7018	904	12.9
Pancreaticoduodenectomy	2011	8305	67	0.8
	2012	9329	121	1.3
	2013	10 068	156	1.5
	2014	10 400	124	1.2
	2015	10 576	53	0.5
	2016	11 028	118	1.1
	2017	11 580	188	1.6
	2018	11 626	194	1.7
	2019	11 813	308	2.6
Acute diffuse peritonitis surgery	2011	7753	488	6.3
	2012	9177	652	7.1
	2013	10 447	1070	10.2
	2014	12 085	1381	11.4
	2015	13 030	1638	12.6
	2016	13 981	2164	15.5
	2017	14 423	2478	17.2
	2018	14 835	2820	19.0
	2019	15 765	3341	21.2

## 4 DISCUSSION

Data of gastroenterological surgery in Japan using the gastroenterological section of the NCD were summarized, and the trends in the 115 gastroenterological procedures and eight major gastroenterological surgeries were reported. The numbers, demography, morbidities, and mortalities of the procedures comprised the main contents of this report, summarized as follows: 1) Operative numbers gradually increased in all procedures except for gastrectomy and hepatectomy, which decreased in these years; 2) age distributions shifted toward older patients in all eight major gastroenterological surgeries; 3) morbidities of ESO, HEP, and PD increased, but the mortalities were minimized in all procedures; 4) laparoscopic procedures have been increasing in all eight major gastroenterological procedures; and 5) the involvement of board‐certified surgeons increased. These trends in recent years were more prominent in 2019.

The 30‐ and 90‐day mortalities have been steadily decreasing in these years, while the postoperative morbidities classified as C–D grade III or higher remained almost unchanged in the gallbladder, rectum, and anus (LAR) and ADP surgery and increased slightly in the esophagus (ESO), stomach and duodenum (DG/TG), small intestine and colon (RHC), liver (HEP), and pancreas (PD). The reason for the discrepancy between postoperative morbidities and mortalities was unclear, but possible reasons include an improved “failure to rescue ratio,”^28^, ^29^ reduction in severe morbidities with higher mortality risk, and more accurate registration of postoperative morbidities into the NCD system than before. These possibilities should be further evaluated in future studies.

The rate of laparoscopic surgeries, including robotic surgeries, demonstrated their rapid increase, reflecting the current trends in gastroenterological surgeries, although the usage rate of laparoscopy varied greatly. Among the major GE procedures, LAR and ESO had the highest rate (more than two‐thirds), while PD had the lowest laparoscopic rate of only 2.6%. Detailed analyses including the safety and other outcomes of laparoscopic procedures should be performed in future studies.

Seven of the eight major gastroenterological surgeries, apart from ADP, can be divided into two categories: 1) basic gastroenterological surgeries (DG/TG, RHC, and LAR) and 2) advanced gastroenterological surgeries (ESO, HEP, and PD). Postoperative complications and mortalities were higher, and the rate of involvement of board‐certified surgeons as the primary surgeons was higher in advanced than in basic gastroenterological surgeries. It is not difficult to imagine that young, noncertified surgeons initially performed basic surgeries and improved their skills, thereafter proceeding to advanced surgeries. Further detailed analyses of the NCD data would clarify this point in the future.

Among the procedures performed in over 50 cases in 2019 in 115 gastroenterological procedures, nine procedures had a mortality of over 10%, and most of these were low‐difficulty procedures, indicating that the causes of mortality were more likely not technical problems but rather the poor general conditions of the patients. In this report, the NCD data between 2011 and 2019 were utilized. It is important to maintain accurate clinical data for analysis and interpretation. To ensure accuracy, systematic audits comparing the registered data and corresponding clinical charts in 20 cases per facility were initiated, and members of the JSGS committee and its subcommittee for NCD database quality improvement investigated 5% of all JSGS‐certified hospitals (about 40 facilities), which were randomly selected every year. This will be continued by the JSGS database committee. The reports of these audits have so far revealed the high accuracy of the NCD data in the JSGS section.^30^, ^31^

The usage of the NCD for clinical studies has been also expanding. JSGS leads the joint council for the society‐specific registries since 2009 and has promoted clinical studies using the NCD. The joint council currently consists of 16 societies as of January 2021; JSGS, JSS, JSHBPS, The Japan Esophageal Society, Japanese Gastric Cancer Association, Japan Pancreas Society, Japan Society for Endoscopic Surgery, Japanese Society of Abdominal Emergency Medicine, Japanese Hernia Society, The Japanese Society for Treatment of Obesity, The Japanese Association for Thoracic Surgery, Japanese Liver Transplantation Society, The Japanese Society for Cancer of the Colon and Rectum, Liver Cancer Study Group of Japan, Endoscopic Liver Surgery Study Group, and Japanese Society for Endoscopic and Robotic Pancreatic Surgery.

The JSGS approved 77 studies from 2013 to 2020 with regard to the joint council, and many high‐impact articles have been published so far. Fifteen articles were accepted and published in 2020, where preoperative risk models for postgastrectomy intraabdominal infectious complications related to gastric cancer,^32^ morbidities after total pancreatectomy,^33^ bile leakage after hepatectomies for hepatocellular carcinoma,^34^ and emergency surgery for gastrointestinal cancer^35^ were reported. The importance of board‐certified surgeons was reported in RHC^36^ and PD.^37^ The Endoscopic Surgical Skill Qualification System certification by the Japan Society for Endoscopic Surgery did not affect the postoperative mortality following laparoscopic DG and LAR.^38^ Additionally, hospital volume affected postoperative mortality after TG^39^ and PD,^40^ and laparoscopic liver resection was safely developed with a low mortality and complication rate relative to open liver resection in Japan.^41^ A geriatric surgery pilot study was conducted from 2017 to 2020, and the specific variables and outcome predictors in geriatric surgery were implemented in the NCD system in 2021.^42^ Thus, in the NCD, a robust nationwide registry on surgical outcomes is important to elucidate the performance of surgeries, to provide tools for future studies, and to improve the surgical outcomes.

The database itself is only a result of the clinical treatment, but it is important for establishing the four pillars of surgical quality improvement that the American College of Surgeons–National Surgical Quality Improvement Program (ACS‐NSQIP) has identified.^43^ These four pillars are setting standards, creating the infrastructure required to achieve these standards, commitment to measuring performance against those standards and remaining accountable for those measurements, and agreeing to a peer review against those standards. The JSGS and ACS‐NSQIP have collaborated since the foundation of the NCD in 2010, and collaborative studies are ongoing.^3^, ^44^ Rigorous data collection is required with respect to the third pillar. While the other pillars are important, establishing standards could be the essential step.

Besides the data presented in this annual report, many other variables were also available, and future studies are expected to elucidate the current situation and implications for the future. Further active clinical studies will discover new evidence using the assets of the NCD data, which all surgeons, medical staff, and surgical clinical reviewers contributed to, in most facilities in Japan. We continuously take care to promote the value of the database and to encourage the usage of feedback and clinical studies using the NCD now and in the future.

## ETHICAL APPROVAL

The protocol for this research project has been approved by the Ethics Committee of the NCD as of November 18, 2020, and it conforms to the provisions of the Declaration of Helsinki as revised in Fortaleza, Brazil, October 2013. The opt‐out method to obtain patient consent was utilized at each institution.

## DISCLOSURE

Funding: The department is a social collaboration department supported by grants from the National Clinical Database, Johnson & Johnson KK, and Nipro Co.

CONFLICT OF INTEREST

Arata Takahashi, Hiroyuki Yamamoto, and Hiroaki Miyata are affiliated with the Department of Healthcare Quality Assessment at the University of Tokyo. The other authors have no conflicts of interest.

## Supporting information

Table S1
