# Supplementary material for: Surgical outcomes in gastroenterological surgery in Japan: Report of the National Clinical Database 2011–2019
Source: Ann Gastroenterol Surg. 2021 Apr 9;5(5):639–58. doi: 10.1002/ags3.12462 (PMC8452469; doi:10.1002/ags3.12462)
Supplement: Supplementary file 1 — Table S1 [file AGS3-5-639-s001.docx]

**Supplemental Table 1-1** Number of surgeries, endoscopic surgery, and mortality rates according to the selected 115 gastrointestinal operative procedures in 2019 (esophagus)

| Organ | Degree of difficulty | Procedure | No. surgeries | **No. Endoscopic  surgeries/ rate (%)** | | | No. postoperative complications* /rate (%) | | | No. postoperative 30-day mortalities /rate (%) | | | No. postoperative 90-day mortalities /rate (%) | | |
| --- | --- | --- | --- | --- | --- | --- | --- | --- | --- | --- | --- | --- | --- | --- | --- |
| Esophagus | Low | Cervical periesophageal abscess drainage | 37 | 7 | / | 18.9 | 14 | / | 37.8 | 1 | / | 2.7 | 2 | / | 5.4 |
|  | Med | Esophageal suture (perforation, injury) | 220 | 23 | / | 10.5 | 70 | / | 31.8 | 10 |  | 4.5 | 15 |  | 6.8 |
|  | Med | Thoracic periesophageal abscess drainage | 24 | 2 | / | 8.3 | 10 | / | 41.7 | 2 |  | 8.3 | 2 |  | 8.3 |
|  | Med | Esophageal foreign body extraction | 24 | 5 | / | 20.8 | 3 | / | 12.5 | 0 |  | 0.0 | 0 |  | 0.0 |
|  | Med | Esophageal diverticulum resection | 47 | 15 | / | 31.9 | 4 | / | 8.5 | 0 |  | 0.0 | 0 |  | 0.0 |
|  | Med | Benign esophageal tumor removal | 65 | 45 | / | 69.2 | 5 | / | 7.7 | 1 |  | 1.5 | 2 |  | 3.1 |
|  | Med | Esophageal resection (removal only) | 671 | 402 | / | 59.9 | 103 | / | 15.4 | 13 |  | 1.9 | 26 |  | 3.9 |
|  | Med | Esophageal reconstruction: reconstruction only (gastric tube reconstruction) | 583 | 359 | / | 61.6 | 109 | / | 18.7 | 6 |  | 1.0 | 18 |  | 3.1 |
|  | Med | Esophageal fistula construction | 196 | 73 | / | 37.2 | 71 | / | 36.2 | 12 |  | 6.1 | 25 |  | 12.8 |
|  | Med | Esophagocardioplasty | 284 | 201 | / | 70.8 | 21 | / | 7.4 | 4 |  | 1.4 | 6 |  | 2.1 |
|  | Med | Achalasia surgery | 225 | 184 | / | 81.8 | 3 | / | 1.3 | 0 |  | 0.0 | 0 |  | 0.0 |
|  | High | Esophagectomy | 6298 | 4209 | / | 66.8 | 1435 | / | 22.8 | 54 |  | 0.9 | 120 |  | 1.9 |
|  | High | Esophageal reconstruction: reconstruction only (colon reconstruction) | 35 | 16 | / | 45.7 | 9 | / | 25.7 | 1 |  | 2.9 | 2 |  | 5.7 |
|  | High | Esophageal bypass | 141 | 27 | / | 19.1 | 51 | / | 36.2 | 10 |  | 7.1 | 19 |  | 13.5 |
|  | High | Bronchoesophageal fistula surgery | 7 | 0 | / | 0.0 | 4 | / | 57.1 | 1 |  | 14.3 | 1 |  | 14.3 |
|  | High | Secondary esophageal reconstruction | 367 | 30 | / | 8.2 | 123 | / | 33.5 | 4 |  | 1.1 | 8 |  | 2.2 |

**Supplemental Table 1-2** Number of surgeries, endoscopic surgery, and mortality rates according to the selected 115 gastrointestinal operative procedures in 2019 (stomach and duodenum）

| Organ | Degree of difficulty | Procedure | No. surgeries | **No. Endoscopic  surgeries/ rate (%)** | | | No. postoperative complications* /rate (%) | | | No. postoperative 30-day mortalities /rate (%) | | | No. postoperative 90-day mortalities /rate (%) | | |
| --- | --- | --- | --- | --- | --- | --- | --- | --- | --- | --- | --- | --- | --- | --- | --- |
| Stomach and duodenum | Low | Gastrostomy and suture gastrorrhaphy | 63 | 5 | / | 7.9 | 5 | / | 7.9 | 1 | / | 1.6 | 1 | / | 1.6 |
|  | Low | Diverticulum，polypectomy (excluding endoscopic resection) | 142 | 22 | / | 15.5 | 11 | / | 7.7 | 2 | / | 1.4 | 2 | / | 1.4 |
|  | Low | Truncal vagotomy | 1 | 1 | / | 100.0 | 0 | / | 0.0 | 0 | / | 0.0 | 0 | / | 0.0 |
|  | Low | Gastroenterostomy (Including duodenal jejunostomy) | 5931 | 1623 | / | 27.4 | 944 | / | 15.9 | 266 | / | 4.5 | 537 | / | 9.1 |
|  | Low | Gastric fistula construction (Excluding PEG) | 1671 | 278 | / | 16.6 | 352 | / | 21.1 | 102 | / | 6.1 | 194 | / | 11.6 |
|  | Low | Gastric pyloroplasty | 86 | 17 | / | 19.8 | 3 | / | 3.5 | 1 | / | 1.2 | 2 | / | 2.3 |
|  | Low | Gastric volvulus (volvulus) surgery and rectopexy | 75 | 41 | / | 54.7 | 5 | / | 6.7 | 0 | / | 0.0 | 0 | / | 0.0 |
|  | Low | Gastric suture (including gastric suture for gastric rupture，Suture closure for gastroduodenal perforation，omental implantation and omental transposition） | 5552 | 1944 | / | 35.0 | 923 | / | 16.6 | 242 | / | 4.4 | 375 | / | 6.8 |
|  | Low | Local gastrectomy (including wedge resection)） | 4707 | 3361 | / | 71.4 | 148 | / | 3.1 | 19 | / | 0.4 | 30 | / | 0.6 |
|  | Med | Gastrectomy (including distal gastrectomy, pylorus preserving gastrectomy and segmental [transverse] gastrectomy) | 33177 | 17205 | / | 51.9 | 2361 | / | 7.1 | 253 | / | 0.8 | 427 | / | 1.3 |
|  | Med | Selective vagotomy | 14 | 10 | / | 71.4 | 0 | / | 0.0 | 0 | / | 0.0 | 0 | / | 0.0 |
|  | High | Total gastrectomy (including fundusectomy)） | 12188 | 3351 | / | 27.5 | 1406 | / | 11.5 | 136 | / | 1.1 | 258 | / | 2.1 |
|  | High | Left upper abdominal exenteration | 3 | 1 | / | 33.3 | 1 | / | 33.3 | 0 | / | 0.0 | 0 | / | 0.0 |

**Supplemental Table 1-3** Number of surgeries, endoscopic surgery, and mortality rates according to the selected 115 gastrointestinal operative procedures in 2019 (small intestine and colon)

| Organ | Degree of difficulty | Procedure | No. surgeries | **No. Endoscopic  surgeries/ rate (%)** | | | No. postoperative complications* /rate (%) | | | No. postoperative 30-day mortalities /rate (%) | | | No. postoperative 90-day mortalities /rate (%) | | |
| --- | --- | --- | --- | --- | --- | --- | --- | --- | --- | --- | --- | --- | --- | --- | --- |
| Small intestine and colon | Low | Enterotomy and enterorrhaphy | 4395 | 686 | / | 15.6 | 809 | / | 18.4 | 191 | / | 4.3 | 393 | / | 8.9 |
|  | Low | Disinvagination (invasive) | 218 | 48 | / | 22.0 | 16 | / | 7.3 | 1 | / | 0.5 | 6 | / | 2.8 |
|  | Low | Partial enterectomy (benign) | 9261 | 1677 | / | 18.1 | 1887 | / | 20.4 | 623 | / | 6.7 | 863 | / | 9.3 |
|  | Low | Ileocecal resection (benign) | 4670 | 1913 | / | 41.0 | 467 | / | 10.0 | 103 | / | 2.2 | 154 | / | 3.3 |
|  | Low | Partial colectomy and sigmoid colectomy (benign) | 8161 | 2544 | / | 31.2 | 1245 | / | 15.3 | 322 | / | 3.9 | 441 | / | 5.4 |
|  | Low | Appendectomy | 56209 | 38984 | / | 69.4 | 1027 | / | 1.8 | 68 | / | 0.1 | 107 | / | 0.2 |
|  | Low | Enterostomy and closure (without enterectomy) | 26837 | 9241 | / | 34.4 | 4593 | / | 17.1 | 1064 | / | 4.0 | 1860 | / | 6.9 |
|  | Med | Enterectomy (malignant) | 3624 | 985 | / | 27.2 | 443 | / | 12.2 | 93 | / | 2.6 | 151 | / | 4.2 |
|  | Med | Ileocecal resection (malignant) | 14794 | 9050 | / | 61.2 | 774 | / | 5.2 | 95 | / | 0.6 | 155 | / | 1.0 |
|  | Med | Partial colectomy and sigmoid colectomy (malignant) | 31901 | 19546 | / | 61.3 | 2075 | / | 6.5 | 207 | / | 0.6 | 340 | / | 1.1 |
|  | Med | Right hemicolectomy | 22410 | 11769 | / | 52.5 | 1666 | / | 7.4 | 306 | / | 1.4 | 449 | / | 2.0 |
|  | Med | Left hemicolectomy | 6227 | 3141 | / | 50.4 | 663 | / | 10.6 | 111 | / | 1.8 | 167 | / | 2.7 |
|  | Med | Total colectomy | 1601 | 489 | / | 30.5 | 385 | / | 24.0 | 145 | / | 9.1 | 187 | / | 11.7 |
|  | Med | Intestinal obstruction surgery (with bowel resection) | 25067 | 5784 | / | 23.1 | 2568 | / | 10.2 | 585 | / | 2.3 | 873 | / | 3.5 |
|  | Med | Enterostomy and closure (with enterectomy) | 23854 | 4114 | / | 17.2 | 3400 | / | 14.3 | 755 | / | 3.2 | 1149 | / | 4.8 |
|  | High | Proctocolectomy and ileoanal (canal) anastomosis | 383 | 191 | / | 49.9 | 43 | / | 11.2 | 2 | / | 0.5 | 3 | / | 0.8 |

**Supplemental Table 1-4** Number of surgeries, endoscopic surgery, and mortality rates according to the selected 115 gastrointestinal operative procedures in 2019 (rectum and anus)

| Organ | Degree of difficulty | Procedure | No. surgeries | **No. Endoscopic  surgeries/ rate (%)** | | | No. postoperative complications* /rate (%) | | | No. postoperative 30-day mortalities /rate (%) | | | No. postoperative 90-day mortalities /rate (%) | | |
| --- | --- | --- | --- | --- | --- | --- | --- | --- | --- | --- | --- | --- | --- | --- | --- |
| Rectum | Low | Transanal rectal tumor removal | 3858 | 97 | / | 2.5 | 23 | / | 0.6 | 3 | / | 0.1 | 5 | / | 0.1 |
|  | Low | Proctocele surgery (transanal) | 2698 | 20 | / | 0.7 | 46 | / | 1.7 | 5 | / | 0.2 | 12 | / | 0.4 |
|  | Med | Rectectomy (benign) | 1237 | 107 | / | 8.6 | 243 | / | 19.6 | 29 | / | 2.3 | 45 | / | 3.6 |
|  | Med | High anterior resection | 11193 | 7761 | / | 69.3 | 723 | / | 6.5 | 72 | / | 0.6 | 97 | / | 0.9 |
|  | Med | Hartmann’s procedure | 6355 | 1197 | / | 18.8 | 1338 | / | 21.1 | 337 | / | 5.3 | 482 | / | 7.6 |
|  | Med | Proctocele surgery (abdominoperineal) | 2044 | 1151 | / | 56.3 | 41 | / | 2.0 | 4 | / | 0.2 | 9 | / | 0.4 |
|  | Med | Malignant anorectal tumor excision (transanal) | 737 | 90 | / | 12.2 | 36 | / | 4.9 | 5 | / | 0.7 | 11 | / | 1.5 |
|  | Med | Anal sphincteroplasty (by tissue replacement) | 2687 | 19 | / | 0.7 | 16 | / | 0.6 | 2 | / | 0.1 | 3 | / | 0.1 |
|  | High | Rectectomy (malignant) | 5163 | 3527 | / | 68.3 | 653 | / | 12.6 | 29 | / | 0.6 | 49 | / | 0.9 |
|  | High | Low anterior resection | 21262 | 14950 | / | 70.3 | 2320 | / | 10.9 | 73 | / | 0.3 | 119 | / | 0.6 |
|  | High | Pelvic evisceration | 437 | 99 | / | 22.7 | 132 | / | 30.2 | 4 | / | 0.9 | 7 | / | 1.6 |
|  | High | Anorectal malignant tumor excision (posterior approach) | 35 | 3 | / | 8.6 | 2 | / | 5.7 | 0 | / | 0.0 | 0 | / | 0.0 |

**Supplemental Table 1-5** Number of surgeries, endoscopic surgery, and mortality rates according to the selected 115 gastrointestinal operative procedures in 2019 (liver)

| Organ | Degree of difficulty | Procedure | No. surgeries | **No. Endoscopic  surgeries/ rate (%)** | | | No. postoperative complications* /rate (%) | | | No. postoperative 30-day mortalities /rate (%) | | | No. postoperative 90-day mortalities /rate (%) | | |
| --- | --- | --- | --- | --- | --- | --- | --- | --- | --- | --- | --- | --- | --- | --- | --- |
| Liver | Low | Hepatorrhaphy | 50 | 3 | / | 6.0 | 15 | / | 30.0 | 5 | / | 10.0 | 5 | / | 10.0 |
|  | Low | Liver abscess drainage (excluding percutaneous procedures) | 47 | 7 | / | 14.9 | 8 | / | 17.0 | 1 | / | 2.1 | 3 | / | 6.4 |
|  | Low | Hepatic cyst resection. Suture. Drainage | 898 | 680 | / | 75.7 | 29 | / | 3.2 | 0 | / | 0.0 | 3 | / | 0.3 |
|  | Low | Partial hepatectomy | 12799 | 4696 | / | 36.7 | 853 | / | 6.7 | 51 | / | 0.4 | 82 | / | 0.6 |
|  | Low | Liver biopsy (excluding percutaneous procedures) | 305 | 35 | / | 11.5 | 22 | / | 7.2 | 1 | / | 0.3 | 14 | / | 4.6 |
|  | Low | Liver coagulonecrotic therapy (excluding percutaneous procedures) | 584 | 126 | / | 21.6 | 40 | / | 6.8 | 4 | / | 0.7 | 5 | / | 0.9 |
|  | Med | Lateral segmentectomy of the liver | 1494 | 559 | / | 37.4 | 82 | / | 5.5 | 8 | / | 0.5 | 12 | / | 0.8 |
|  | Med | Esophageal and gastric varix surgery | 31 | 17 | / | 54.8 | 7 | / | 22.6 | 2 | / | 6.5 | 3 | / | 9.7 |
|  | High | Hepatectomy (segmented or more; excluding lateral segments) | 7018 | 904 | / | 12.9 | 1058 | / | 15.1 | 94 | / | 1.3 | 143 | / | 2.0 |
|  | High | Systematic subsegmentectomy | 2527 | 571 | / | 22.6 | 240 | / | 9.5 | 8 | / | 0.3 | 22 | / | 0.9 |
|  | High | Liver transplant | 685 | 5 | / | 0.7 | 194 | / | 28.3 | 18 | / | 2.6 | 31 | / | 4.5 |
|  | High | Hepatopancreatoduodenectomy | 144 | 2 | / | 1.4 | 76 | / | 52.8 | 9 | / | 6.3 | 11 | / | 7.6 |

**Supplemental Table 1-6** Number of surgeries, endoscopic surgery, and mortality rates according to the selected 115 gastrointestinal operative procedures in 2019 (gall bladder)

| Organ | Degree of difficulty | Procedure | No. surgeries | **No. Endoscopic  surgeries/ rate (%)** | | | No. postoperative complications* /rate (%) | | | No. postoperative 30-day mortalities /rate (%) | | | No. postoperative 90-day mortalities /rate (%) | | |
| --- | --- | --- | --- | --- | --- | --- | --- | --- | --- | --- | --- | --- | --- | --- | --- |
| Gall bladder | Low | Cholangiotomy | 72 | 8 | / | 11.1 | 16 | / | 22.2 | 2 | / | 2.8 | 3 | / | 4.2 |
|  | Low | Cysticolithectomy | 68 | 19 | / | 27.9 | 8 | / | 11.8 | 2 | / | 2.9 | 2 | / | 2.9 |
|  | Low | Cholecystectomy | 133265 | 93797 | / | 70.4 | 4623 | / | 3.5 | 437 | / | 0.3 | 729 | / | 0.5 |
|  | Low | External cholecystostomy | 113 | 13 | / | 11.5 | 26 | / | 23.0 | 7 | / | 6.2 | 12 | / | 10.6 |
|  | Low | Cystoenteric anastomosis | 43 | 4 | / | 9.3 | 6 | / | 14.0 | 2 | / | 4.7 | 5 | / | 11.6 |
|  | Med | Cysticolithectomy | 2483 | 642 | / | 25.9 | 239 | / | 9.6 | 32 | / | 1.3 | 53 | / | 2.1 |
|  | Med | Biliary tract reconstruction | 326 | 20 | / | 6.1 | 76 | / | 23.3 | 8 | / | 2.5 | 12 | / | 3.7 |
|  | Med | Biliary bypass | 1170 | 53 | / | 4.5 | 184 | / | 15.7 | 25 | / | 2.1 | 50 | / | 4.3 |
|  | Med | Cholangioplasty | 118 | 11 | / | 9.3 | 24 | / | 20.3 | 2 | / | 1.7 | 4 | / | 3.4 |
|  | Med | Duodenal papilloplasty | 33 | 2 | / | 6.1 | 11 | / | 33.3 | 1 | / | 3.0 | 2 | / | 6.1 |
|  | Med | Choledocal dilatation | 279 | 58 | / | 20.8 | 25 | / | 9.0 | 0 | / | 0.0 | 0 | / | 0.0 |
|  | Med | Biliary fistula closure | 35 | 9 | / | 25.7 | 6 | / | 17.1 | 1 | / | 2.9 | 1 | / | 2.9 |
|  | High | Malignant gallbladder tumor surgery (excluding simple cholecystectomy)） | 1075 | 53 | / | 4.9 | 120 | / | 11.2 | 3 | / | 0.3 | 4 | / | 0.4 |
|  | High | Malignant bile duct tumor surgery | 1122 | 15 |  | 1.3 | 383 |  | 34.1 | 43 |  | 3.8 | 58 |  | 5.2 |
|  | High | Biliary atresia surgery | 12 | 5 |  | 41.7 | 1 |  | 8.3 | 0 |  | 0.0 | 0 |  | 0.0 |

**Supplemental Table 1-7** Number of surgeries, endoscopic surgery, and mortality rates according to the selected 115 gastrointestinal operative procedures in 2019 (pancreas)

| Organ | Degree of difficulty | Procedure | No. surgeries | **No. Endoscopic  surgeries/ rate (%)** | | | No. postoperative complications* /rate (%) | | | No. postoperative 30-day mortalities /rate (%) | | | No. postoperative 90-day mortalities /rate (%) | | |
| --- | --- | --- | --- | --- | --- | --- | --- | --- | --- | --- | --- | --- | --- | --- | --- |
| Pancreas | Low | External pancreatic cyst drainage | 20 | 3 | / | 15.0 | 12 | / | 60.0 | 3 | / | 15.0 | 5 | / | 25.0 |
|  | Low | External pancreatic duct drainage | 6 | 1 | / | 16.7 | 0 | / | 0.0 | 0 | / | 0.0 | 0 | / | 0.0 |
|  | Med | Pancreatorrhaphy | 4 | 0 | / | 0.0 | 2 | / | 50.0 | 1 | / | 25.0 | 1 | / | 25.0 |
|  | Med | Partial pancreatic resection | 184 | 44 | / | 23.9 | 45 | / | 24.5 | 1 | / | 0.5 | 2 | / | 1.1 |
|  | Med | Distal pancreatectomy (benign) | 1545 | 666 | / | 43.1 | 290 | / | 18.8 | 11 | / | 0.7 | 15 | / | 1.0 |
|  | Med | Pancreatoenteric anastomosis | 25 | 1 | / | 4.0 | 5 | / | 20.0 | 2 | / | 8.0 | 2 | / | 8.0 |
|  | Med | Pancreatic (duct) anastomosis | 252 | 2 | / | 0.8 | 42 | / | 16.7 | 2 | / | 0.8 | 6 | / | 2.4 |
|  | Med | Acute pancreatitis surgery | 69 | 6 | / | 8.7 | 29 | / | 42.0 | 10 | / | 14.5 | 16 | / | 23.2 |
|  | Med | Pancreatolithiasis surgery | 15 | 1 | / | 6.7 | 4 | / | 26.7 | 0 | / | 0.0 | 0 | / | 0.0 |
|  | Med | Plexus pancreaticus capitalis resection | 0 | 0 | / | 0 | 0 | / | 0 | 0 | / | 0 | 0 | / | 0 |
|  | High | Pancreaticoduodenectomy | 11813 | 308 | / | 2.6 | 2854 | / | 24.2 | 119 | / | 1.0 | 210 | / | 1.8 |
|  | High | Distal pancreatectomy (malignant)） | 4912 | 1035 | / | 21.1 | 1038 | / | 21.1 | 22 | / | 0.4 | 44 | / | 0.9 |
|  | High | Total pancreatectomy | 587 | 3 | / | 0.5 | 102 | / | 17.4 | 25 | / | 4.3 | 31 | / | 5.3 |
|  | High | Duodenum preserving pancreas head resection | 62 | 1 | / | 1.6 | 17 | / | 27.4 | 0 | / | 0.0 | 0 | / | 0.0 |
|  | High | Segmental pancreatic resection | 162 | 10 | / | 6.2 | 66 | / | 40.7 | 1 | / | 0.6 | 1 | / | 0.6 |
|  | HIgh | Distal pancreatectomy | 47 | 0 | / | 0.0 | 16 | / | 34.0 | 2 | / | 4.3 | 2 | / | 4.3 |

**Supplemental Table 1-8** Annual changes in the number of surgeries according to the selected 115 gastrointestinal operative procedures in 2019 (spleen)

| Organ | Degree of difficulty | Procedure | No. surgeries | **No. Endoscopic  surgeries/ rate (%)** | | | No. postoperative complications* /rate (%) | | | No. postoperative 30-day mortalities /rate (%) | | | No. postoperative 90-day mortalities /rate (%) | | |
| --- | --- | --- | --- | --- | --- | --- | --- | --- | --- | --- | --- | --- | --- | --- | --- |
| Spleen | Low | Splenorrhaphy | 32 | 2 | / | 6.3 | 6 | / | 18.8 | 1 | / | 3.1 | 1 | / | 3.1 |
|  | Med | Splenectomy | 2354 | 695 | / | 29.5 | 372 | / | 15.8 | 70 | / | 3.0 | 96 | / | 4.1 |
|  | Med | Partial splenic resection | 27 | 11 | / | 40.7 | 2 | / | 7.4 | 0 | / | 0.0 | 0 | / | 0.0 |

**Supplemental Table 1-9** Annual changes in the number of surgeries according to the selected 115 gastrointestinal operative procedures in 2019 (other)

| Organ | Degree of difficulty | Procedure | No. surgeries | **No. Endoscopic  surgeries/ rate (%)** | | | No. postoperative complications* /rate (%) | | | No. postoperative 30-day mortalities /rate (%) | | | No. postoperative 90-day mortalities /rate (%) | | |
| --- | --- | --- | --- | --- | --- | --- | --- | --- | --- | --- | --- | --- | --- | --- | --- |
| Other | Low | Localized intra-abdominal abscess surgery | 2421 | 710 | / | 29.3 | 382 | / | 15.8 | 55 | / | 2.3 | 104 | / | 4.3 |
|  | Low | Exploratory laparotomy | 12542 | 5903 | / | 47.1 | 1975 | / | 15.7 | 767 | / | 6.1 | 1069 | / | 8.5 |
|  | Med | Acute diffuse peritonitis surgery | 15765 | 3341 | / | 21.2 | 4367 | / | 27.7 | 1233 | / | 7.8 | 1795 | / | 11.4 |
|  | Med | Ventral hernia surgery | 14384 | 4278 | / | 29.7 | 475 | / | 3.3 | 64 | / | 0.4 | 111 | / | 0.8 |
|  | Med | Diaphragm suture | 269 | 85 | / | 31.6 | 46 | / | 17.1 | 10 | / | 3.7 | 12 | / | 4.5 |
|  | Med | Esophageal hiatus hernia surgery | 1205 | 824 | / | 68.4 | 88 | / | 7.3 | 12 | / | 1.0 | 23 | / | 1.9 |
|  | Med | Retroperitoneal tumor surgery | 1529 | 132 | / | 8.6 | 123 | / | 8.0 | 6 | / | 0.4 | 15 | / | 1.0 |
|  | Med | Abdominal wall/mesenteric/omental tumor resection | 1920 | 537 | / | 28.0 | 162 | / | 8.4 | 31 | / | 1.6 | 39 | / | 2.0 |
|  | Med | Gastrointestinal perforation closure | 428 | 55 | / | 12.9 | 128 | / | 29.9 | 42 | / | 9.8 | 51 | / | 11.9 |
|  | High | Diaphragmatic hiatus hernia surgery | 62 | 20 | / | 32.3 | 4 | / | 6.5 | 1 | / | 1.6 | 1 | / | 1.6 |
